# Supplementary figures and images for: An integrative approach identifies direct targets of the late viral transcription complex and an expanded promoter recognition motif in Kaposi’s sarcoma-associated herpesvirus
Source: PLoS Pathog. 2019 May 16;15(5):e1007774. doi: 10.1371/journal.ppat.1007774 (PMC6541308; doi:10.1371/journal.ppat.1007774)

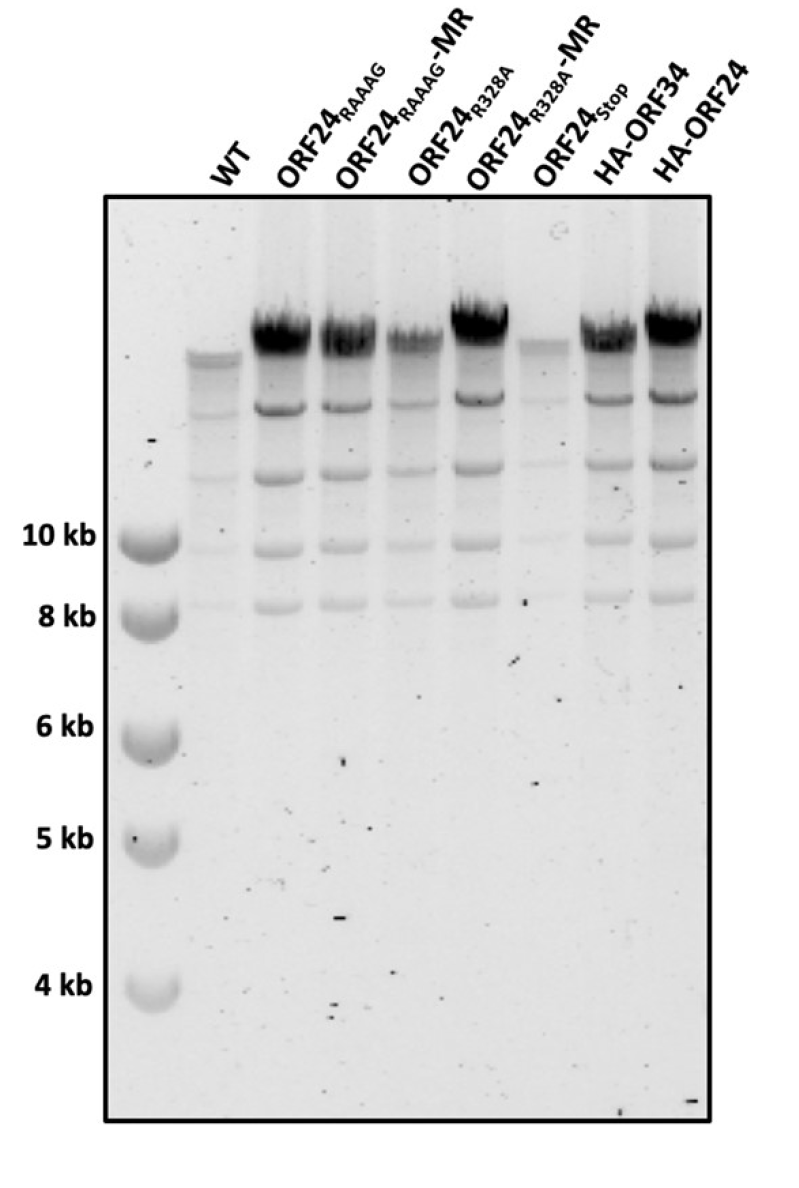

Supplement: S1 Fig — The integrity of the various BAC mutants used in this study was verified by RsrII digestion. (TIF) [file ppat.1007774.s001.tif]

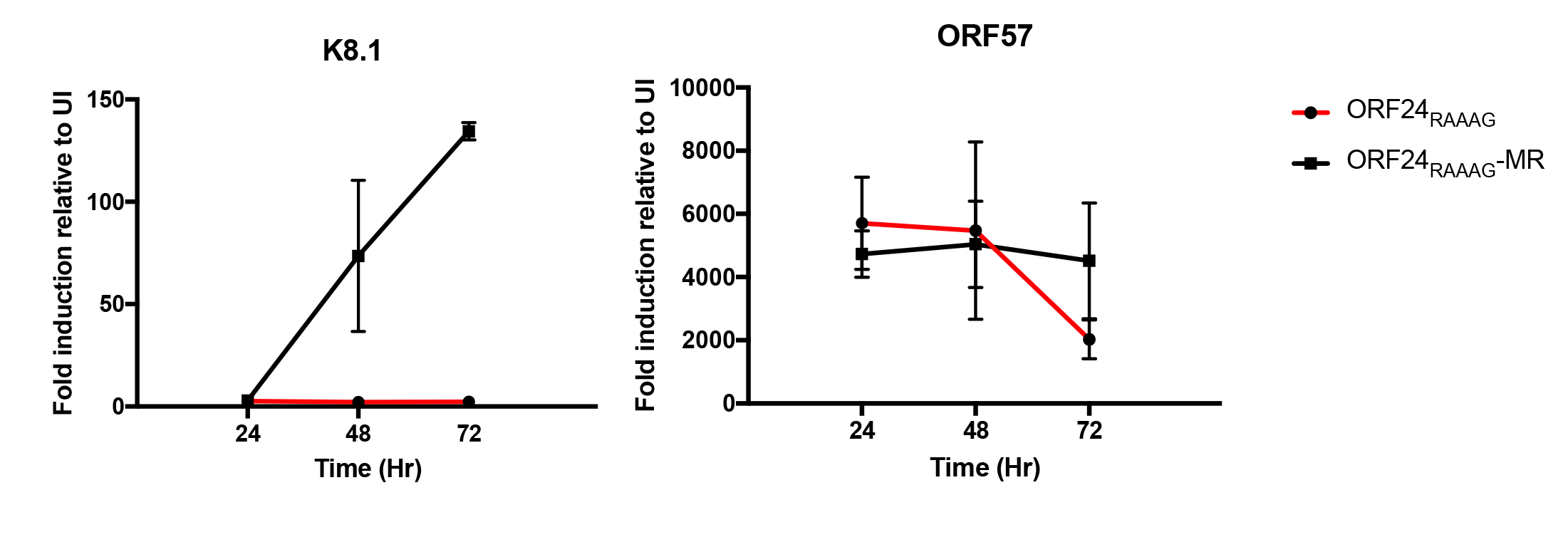

Supplement: S2 Fig — iSLK-ORF24RAAAG and MR were reactivated with doxycycline and sodium butyrate for the indicated time points, whereupon total RNA was extracted, converted to cDNA, and quantified by qPCR using primers specific to the CDS of K8.1 (late gene) or ORF57 (early gene). The fold induction was measured relative to uninduced sample. (TIF) [file ppat.1007774.s002.tif]

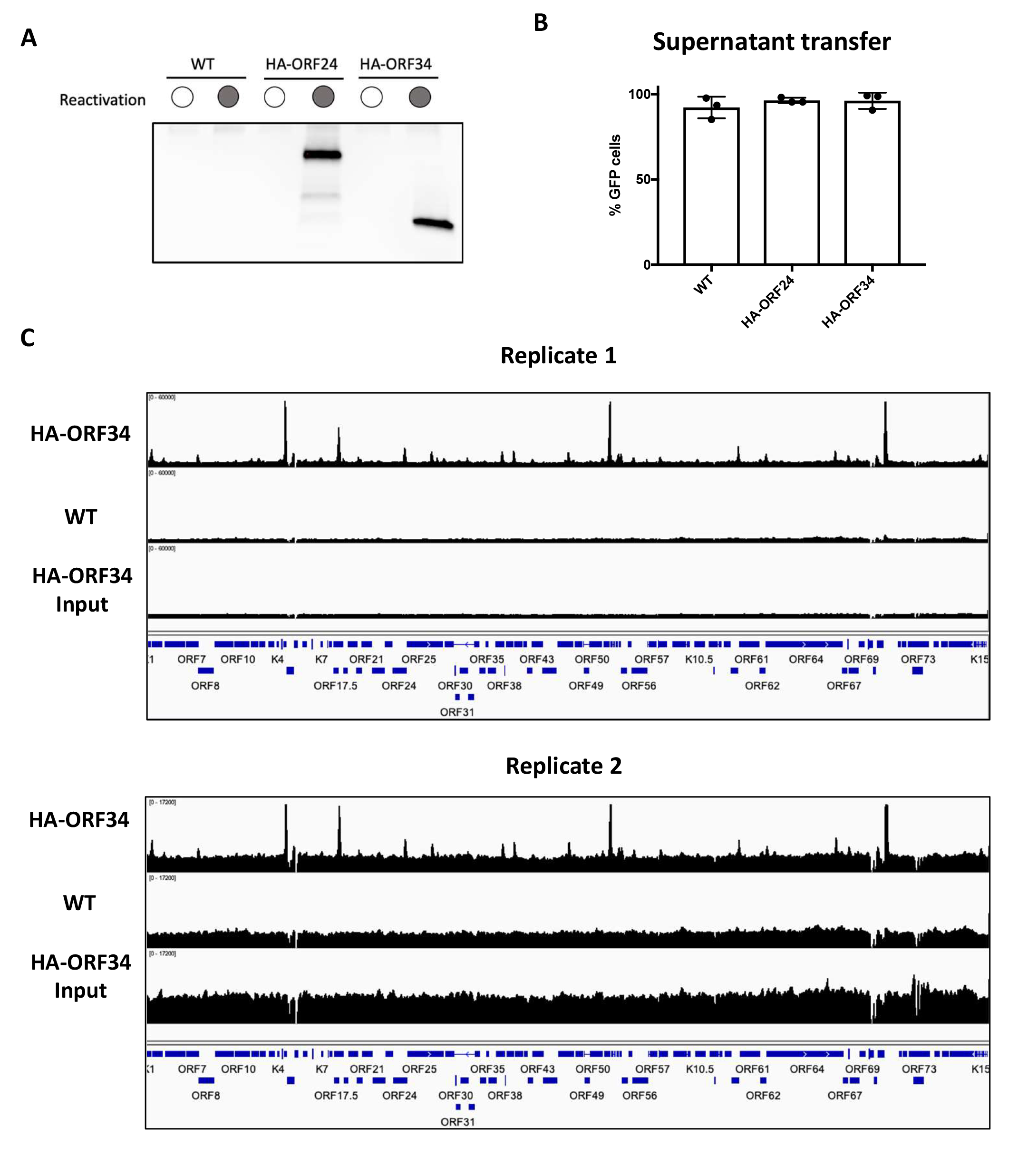

Supplement: S3 Fig — (A) Western blot showing expression of HA-ORF24 and HA-ORF34. iSLK cells harboring WT BAC16, HA-ORF34 BAC or HA-ORF24 BAC were reactivated with doxycycline and sodium butyrate for 72 hours. Protein lysate was immunoprecipitated with HA beads and visualized by SDS-PAGE-Western blot. (B) iSLK cells harboring WT BAC16, HA-ORF34 BAC or HA-ORF24 BAC were reactivated with doxycycline and sodium butyrate for 72 hours. Progeny virion production was measured by supernatant transfer on to 293T cells and quantified by flow cytometry. (B) IGV image showing ChIP-Seq data from iSLK-HA-ORF34 cells reactivated with doxycycline and sodium butyrate for 48 h and immunoprecipitated with anti-HA antibody. Also shown are plots for control data from untagged iSLK cells and input DNA for two biological replicates. (TIF) [file ppat.1007774.s003.tif]

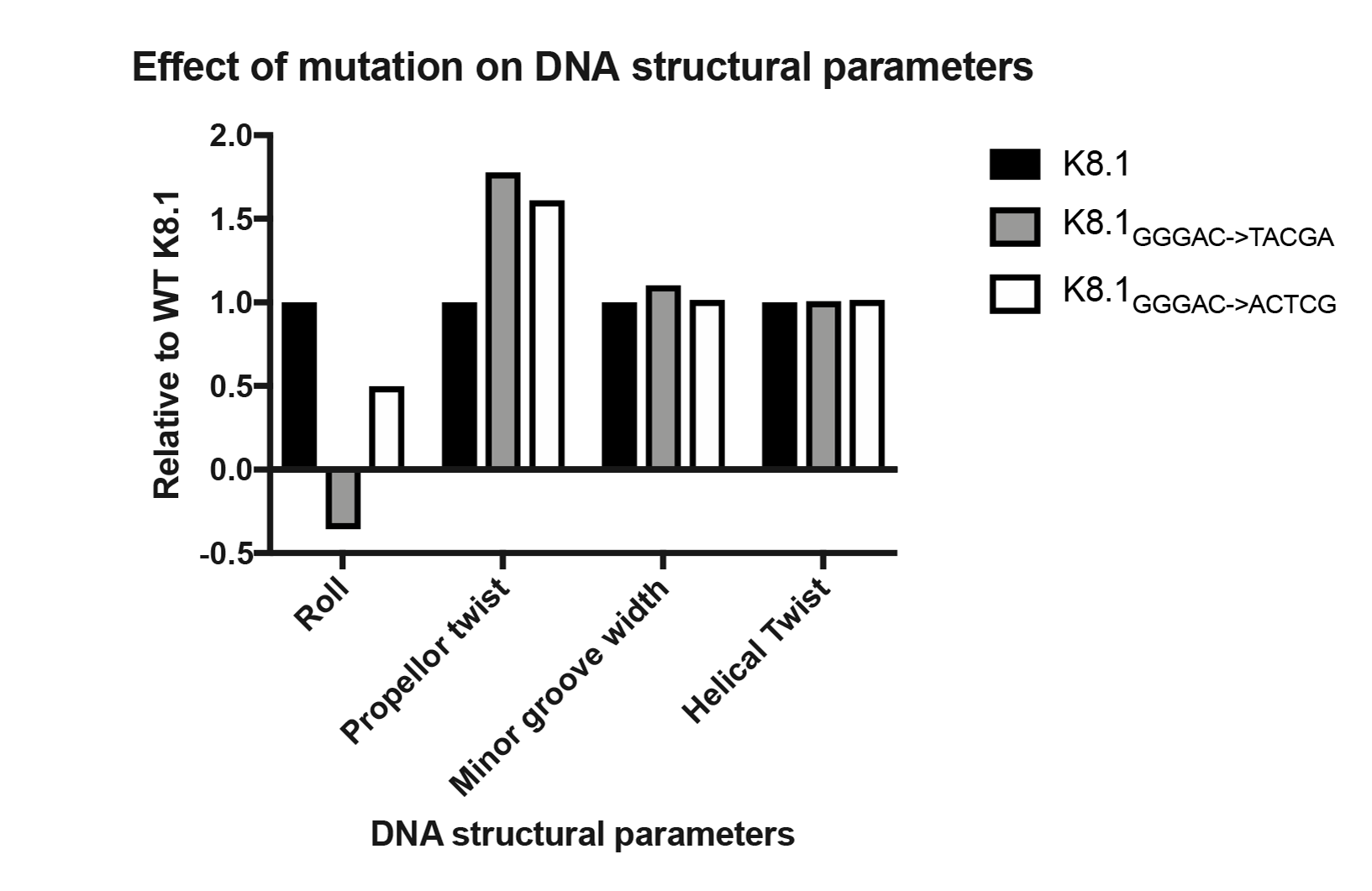

Supplement: S4 Fig — Four structural parameters for 40 bp of the WT and expanded motif mutants were calculated using the DNAShape program in R [49]. Average of each structural parameter for the mutated base pairs and the two adjacent base pairs at either end (a total of 7 bp) was calculated for each DNA sequence analyzed. The two adjacent base pairs were included in the averaging as some of the structural parameters refer to a base step rather than a specific base. The average for each mutant was normalized to the WT K8.1 promoter. (TIF) [file ppat.1007774.s004.tif]
