# Supplementary material for: An integrative approach identifies direct targets of the late viral transcription complex and an expanded promoter recognition motif in Kaposi’s sarcoma-associated herpesvirus
Source: PLoS Pathog. 2019 May 16;15(5):e1007774. doi: 10.1371/journal.ppat.1007774 (PMC6541308; doi:10.1371/journal.ppat.1007774)
Supplement: S1 Table — (PDF) [file ppat.1007774.s005.pdf]

Supplementary Table 1:
List of all DNA sequences used in the study

Name	Sequence	Used for
K8.1Pr_pGL4.16_F	TGGCCTAACTGGCCGGTACCTGTGCCGTCGTCCGGGA	Cloning
K8.1Pr_pGL4.16_R	CCGGATTGCCAAGCTTAGGATTAAGGGATTAACTTCGGTCCC	Cloning
ORF57Pr_pGL4.16_F	TGGCCTAACTGGCCGGTACCAGTGTAACAATAATGTTCCCACGGC	Cloning
ORF57Pr_pGL4.16_R	CCGGATTGCCAAGCTTTTTTGGGAACCTGGCAGCCAGGTTA	Cloning
Orilyt_KSHV_F	GGTCTGACAGCGGCCGCCAGCTTATCCGGTGGCAATCT	Cloning
Orilyt_KSHV_R	AGCACTGGTAACCACTGCAGTGGGCACAGAGATATGTGACGTGACGCCCT	Cloning
K8.1_G8A_1	GGCAGCAATATTAAAAGGACCGAAGTTAATC	Mutagenesis
K8.1_G8A_2	GATTAACTTCGGTCCTTTTAATATTGCTGCC	Mutagenesis
K8.1_G8C_1	GGCAGCAATATTAAACGGACCGAAGTTAATC	Mutagenesis
K8.1_G8C_2	GATTAACTTCGGTCCGTTTAATATTGCTGCC	Mutagenesis
K8.1_G8T_1	GGCAGCAATATTAAATGGACCGAAGTTAATC	Mutagenesis
K8.1_G8T_2	GATTAACTTCGGTCCATTTAATATTGCTGCC	Mutagenesis
K8.1_G9C_1	GCAGCAATATTAAAGCGACCGAAGTTAATCC	Mutagenesis
K8.1_G9C_2	GGATTAACTTCGGTCGCTTTAATATTGCTGC	Mutagenesis
K8.1_G9A_1	GCAGCAATATTAAAGAGACCGAAGTTAATCC	Mutagenesis
K8.1_G9A_2	GGATTAACTTCGGTCTCTTTAATATTGCTGC	Mutagenesis
K8.1_G9T_1	GCAGCAATATTAAAGTGACCGAAGTTAATCC	Mutagenesis
K8.1_G9T_2	GGATTAACTTCGGTCACTTTAATATTGCTGC	Mutagenesis
K8.1_G10T_1	CAGCAATATTAAAGGTACCGAAGTTAATCCC	Mutagenesis
K8.1_G10T_2	GGGATTAACTTCGGTACCTTTAATATTGCTG	Mutagenesis
K8.1_A11C_1	AGCAATATTAAAGGGCCCGAAGTTAATCCCT	Mutagenesis
K8.1_A11C_2	AGGGATTAACTTCGGGCCCTTTAATATTGCT	Mutagenesis
K8.1_A11T_1	AGCAATATTAAAGGGTCCGAAGTTAATCCCT	Mutagenesis
K8.1_A11T_2	AGGGATTAACTTCGGACCCTTTAATATTGCT	Mutagenesis
K8.1_A11G_1	AGCAATATTAAAGGGGCCGAAGTTAATCCCT	Mutagenesis
K8.1_A11G_2	AGGGATTAACTTCGGCCCCTTTAATATTGCT	Mutagenesis
K8.1_C12G_1	GCAATATTAAAGGGAGCGAAGTTAATCCCTT	Mutagenesis
K8.1_C12G_2	AAGGGATTAACTTCGCTCCCTTTAATATTGC	Mutagenesis
K8.1_C12T_1	GCAATATTAAAGGGATCGAAGTTAATCCCTT	Mutagenesis
K8.1_C12T_2	AAGGGATTAACTTCGATCCCTTTAATATTGC	Mutagenesis
K8.1_GG89AC_1	GGCAGCAATATTAAAACGACCGAAGTTAATCCCTT	Mutagenesis
K8.1_GG89AC_2	AAGGGATTAACTTCGGTCGTTTTAATATTGCTGCC	Mutagenesis
K8.1_GG89CT_1	GGCAGCAATATTAAACTGACCGAAGTTAATCCCTT	Mutagenesis
K8.1_GG89CT_2	AAGGGATTAACTTCGGTCAGTTTAATATTGCTGCC	Mutagenesis
K8.1_GG89TT_1	GGCAGCAATATTAAATTGACCGAAGTTAATCCCTT	Mutagenesis
K8.1_GG89TT_2	AAGGGATTAACTTCGGTCAATTTAATATTGCTGCC	Mutagenesis

K8.1_AC1112CG_1	GGCAGCAATATTAAAGGGCGCGAAGTTAATCCCTTAATC	Mutagenesis
K8.1_AC1112CG_2	GATTAAGGGATTAACTTCGCGCCCTTTAATATTGCTGCC	Mutagenesis
K8.1_AC1112GT_1	GGCAGCAATATTAAAGGGGTCGAAGTTAATCCCTTAATC	Mutagenesis
K8.1_AC1112GT_2	GATTAAGGGATTAACTTCGACCCCTTTAATATTGCTGCC	Mutagenesis
K8.1_TATT->TATA_1	GTTATCCGGCAGCAATATAAAAGGGACCGAAGTTAATC	Mutagenesis
K8.1_TATT->TATA_2	GATTAACTTCGGTCCCTTTTATATTGCTGCCGGATAAC	Mutagenesis
K8.1_GGGAC->TACGA_1	GGCAGCAATATTAAATACGACGAAGTTAATCCCTT	Mutagenesis
K8.1_GGGAC->TACGA_2	AAGGGATTAACTTCGTCGTATTTAATATTGCTGCC	Mutagenesis
K8.1_GGGAC->ACTCG_1	GGCAGCAATATTAAAACTCGCGAAGTTAATCCCTT	Mutagenesis
K8.1_GGGAC->ACTCG_2	AAGGGATTAACTTCGCGAGTTTTAATATTGCTGCC	Mutagenesis
K8.1Pr_qPCR_F	GGGAGAACCATGCCAGACTTTG	ChIP-qPCR
K8.1Pr_qPCR_R	GCATAGGATTAGGAGCGCCAC	ChIP-qPCR
ORF59Pr_qPCR_F	AATCCACAGGCATGATTGC	ChIP-qPCR/<br>DNA replication
ORF59Pr_qPCR_R	CACACTTCCACCTCCCCTAA	ChIP-qPCR<br>DNA replication
CPSF6Pr_qPCR_F	CGAGGAATGTCCCTGTTTGT	DNA replication
CPSF6Pr_qPCR_R	ACTGGCTGTCTCCTCTCAGC	DNA replication
K8.1CDS_qPCR_F	CCGTCGGTGTGTAGGGATAAAG	RT-qPCR
K8.1CDS_qPCR_R	GTCGTTGTAGTGGTGGCAGAAA	RT-qPCR
ORF57CDS_qPCR_F	TTTGACGAATCGAGGGACGACG	RT-qPCR
ORF57CDS_qPCR_R	GCAGTTGAGAACGACCTTGAGAT	RT-qPCR
ORF17.5Pr_qPCR_F	GCTGGCCTTTAAATACGCCG	ChIP-qPCR
ORF17.5Pr_qPCR_R	TTTCGTCAGTCCCCTGGAGA	ChIP-qPCR
OriLyt_L_qPCR_F	CGGCGGAGGTTGTCAATAGT	ChIP-qPCR
OriLyt_L_qPCR_R	GGGTCCTGTTCTGGGATAGC	ChIP-qPCR
OriLyt_R_qPCR_F	CCCTGGGCACGATAACCATA	ChIP-qPCR
OriLyt_R_qPCR_R	AGGGGCGCAAAACAATTGTG	ChIP-qPCR
ORF64Pr_qPCR_F	GCAGAAAGAACTTGAGCTCT	ChIP-qPCR
ORF64Pr_qPCR_R	AGGCAGACCGGGAGTACGTT	ChIP-qPCR
ORF37Pr_qPCR_F	GCTGGCGTTTCAGAAGCAGTG	ChIP-qPCR
ORF37Pr_qPCR_R	CCATCCAGGGTGTCAACCA	ChIP-qPCR
K8.1Pr_DoubleMutant_1	GTTATCCGGCAGCAATATAAAATACGACGAAGTTAATCCCTTAATCC	Mutagenesis
K8.1Pr_DoubleMutant_2	GGATTAAGGGATTAACTTCGTCGTATTTTATATTGCTGCCGGATAAC	Mutagenesis
